# The Lhx1-Ldb1 complex interacts with Furry to regulate microRNA expression during pronephric kidney development

**DOI:** 10.1038/s41598-018-34038-x

**Published:** 2018-10-30

**Authors:** Eugenel B. Espiritu, Amanda E. Crunk, Abha Bais, Daniel Hochbaum, Ailen S. Cervino, Yu Leng Phua, Michael B. Butterworth, Toshiyasu Goto, Jacqueline Ho, Neil A. Hukriede, M. Cecilia Cirio

**Affiliations:** 1 Department of Developmental Biology, University of Pittsburgh, Pittsburgh, PA USA; 2 Universidad de Buenos Aires, Departamento de Biodiversidad y Biología Experimental, Buenos Aires, Argentina; 3 Universidad de Buenos Aires, Facultad de Ciencias Exactas y Naturales, Buenos Aires, Argentina; 4 CONICET- Universidad de Buenos Aires, Instituto de Fisiología, Biología Molecular y Neurociencias (IFIBYNE), Buenos Aires, Argentina; 5 Division of Nephrology, Department of Pediatrics, Children’s Hospital of Pittsburgh, University of Pittsburgh, Pittsburgh, PA USA; 6 Department of Cell Biology, University of Pittsburgh, Pittsburgh, PA USA; 7 Department of Molecular Cell Biology, Medical Research Institute, Tokyo Medical and Dental University, Tokyo, Japan; 8 Center for Critical Care Nephrology, University of Pittsburgh, Pittsburgh, PA USA

## Abstract

The molecular events driving specification of the kidney have been well characterized. However, how the initial kidney field size is established, patterned, and proportioned is not well characterized. Lhx1 is a transcription factor expressed in pronephric progenitors and is required for specification of the kidney, but few Lhx1 interacting proteins or downstream targets have been identified. By tandem-affinity purification, we isolated FRY like transcriptional coactivator (Fryl), one of two paralogous genes, *fryl* and *furry* (*fry)*, have been described in vertebrates. Both proteins were found to interact with the Ldb1-Lhx1 complex, but our studies focused on Lhx1/Fry functional roles, as they are expressed in overlapping domains. We found that *Xenopus* embryos depleted of *fry* exhibit loss of pronephric mesoderm, phenocopying the Lhx1*-*depleted animals. In addition, we demonstrated a synergism between Fry and Lhx1, identified candidate microRNAs regulated by the pair, and confirmed these microRNA clusters influence specification of the kidney. Therefore, our data shows that a constitutively-active Ldb1-Lhx1 complex interacts with a broadly expressed microRNA repressor, Fry, to establish the kidney field.

## Introduction

In the vertebrate kidney, the specification of the renal progenitor cell field is required for generating the appropriate number of nephrons, as reduction of the number of renal progenitors results in reduced nephron endowment^1^. Numerous signaling pathways are known to control nephron endowment^2–6^, but how these pathways drive formation of the initial progenitor field is largely unknown. Renal progenitor cells originate from the intermediate mesoderm (IM) and one of the earliest genes expressed throughout the IM is the LIM homeodomain transcription factor, Lhx1^7–11^. Lhx1 plays multiple roles during embryogenesis, including kidney development and establishment of the body axis^7,8,11–15^. During kidney organogenesis, depletion of Lhx1 in *Xenopus* results in loss of the entire pronephric kidney and, in mammals, leads to a complete lack of metanephric kidney^8,11,14,16,17^. Additionally, during zebrafish mesonephric kidney regeneration, *lhx1* becomes reactivated within the self-renewing adult renal progenitor cells that drive neo-nephrogenesis^18^.

Lhx1 regulates the expression of target genes through the formation of multi-protein transcriptional complex^12^. In *Xenopus*, Lhx1 is initially expressed in the organizer, which coordinates anterior-posterior axis formation^15^, and forms a conserved multi-protein complex^12,19–22^. Structurally, Lhx1 contains two LIM domains at the N-terminus, a central homeodomain and five C-terminal conserved regions (CCRs)^12,23^. The LIM domains are zinc-finger motifs that mediate protein-protein interactions, and are thought to repress Lhx1 activity until forming a tetrameric complex with LIM domain-binding (Ldb) proteins^15,24^. Proteins that bind to the Ldb1-Lhx1 complex (i.e. Ssdp1, Otx2, Foxa2), facilitate multiple developmental outcomes^12,22,25–27^. However, none of the interacting partners characterized thus far have been described to play a role during kidney organogenesis.

To identify Lhx1 binding proteins, we performed tandem-affinity purification (TAP) in a *Xenopus* kidney cell line. Among the interacting proteins that bound to a constitutively active Ldb1/Lhx1 chimera was Fryl (FRY like transcriptional co-activator). Two paralogues are found in vertebrates termed *fry* (FRY microtubule binding protein) and *fryl* while *Drosophila*, *Caenorhabditis elegans, Arabidopsis, Saccharomyces* only have *fry*. *Fry* and *fryl* genes are co-expressed in various mouse adult tissues including the spinal cord, brain, and kidney^28^. Fry plays important roles in numerous cellular processes, including cytoskeletal maintenance, cell polarization, cell division, neurite growth and morphogenesis; Fry also acts as activator and scaffold protein of NDR family Ser/Thr kinases^29–34^. However, Fry appears to have NDR independent functions as well, one of them regulating microRNA (miRNA) expression in *Xenopus* axis formation^35^. Less is known about Fryl protein, reported as a transcriptional regulator^36^ and a NOCTH1 transcriptional co-activator^37^. Mice with *fryl* deficiency die soon after birth and survivors present defective metanephric kidney development^28^.

Structurally, Fry and Fryl proteins have a furry domain (FD) that consists of HEAT/Armadillo repeats, and in vertebrates only, contain two leucine-zipper motifs and a coiled-coil structure in the C-terminal region with five less conserved regions in between them^31,35^. The FD and LZ/coiled-coil (LZ) domains are highly conserved among vertebrates and mediate many cellular functions, including repressing miRNA expression^35^.

During tissue patterning and organogenesis, miRNAs control cell fate programs that help define and shape tissue boundaries^38–40^. miRNAs are important regulators of kidney development^5,6,41,42^. In the *Xenopus* pronephros, loss of miRNA biogenesis causes defects in nephron patterning, delayed tubule terminal differentiation, and reduced nephron size^40,43,44^. While there is growing evidence for the role of miRNAs during kidney morphogenesis, little is known about the role of miRNAs in specification of the kidney anlage.

Here, we show that Ldb1-Lhx1 and the functional domains of Fry (FD-LZ) form a protein complex. We demonstrate that embryos depleted of Fry show a loss of the kidney primordium, resembling the phenotype seen in Lhx1-depleted larvae, and identify mature miRNAs with altered levels upon depletion of *lhx1* and *fry*. We also show that Lhx1 and Fry act synergistically to drive specification of the pronephric field as well as to regulate the levels of miRNAs. Lastly, we demonstrate that two of the miRNA clusters affected by the absence of Lhx1/Fry have antagonistic effects on kidney development.

## Results

### Lhx1 interacts with Fryl and functional domains of Fry protein

**Figure 1:**
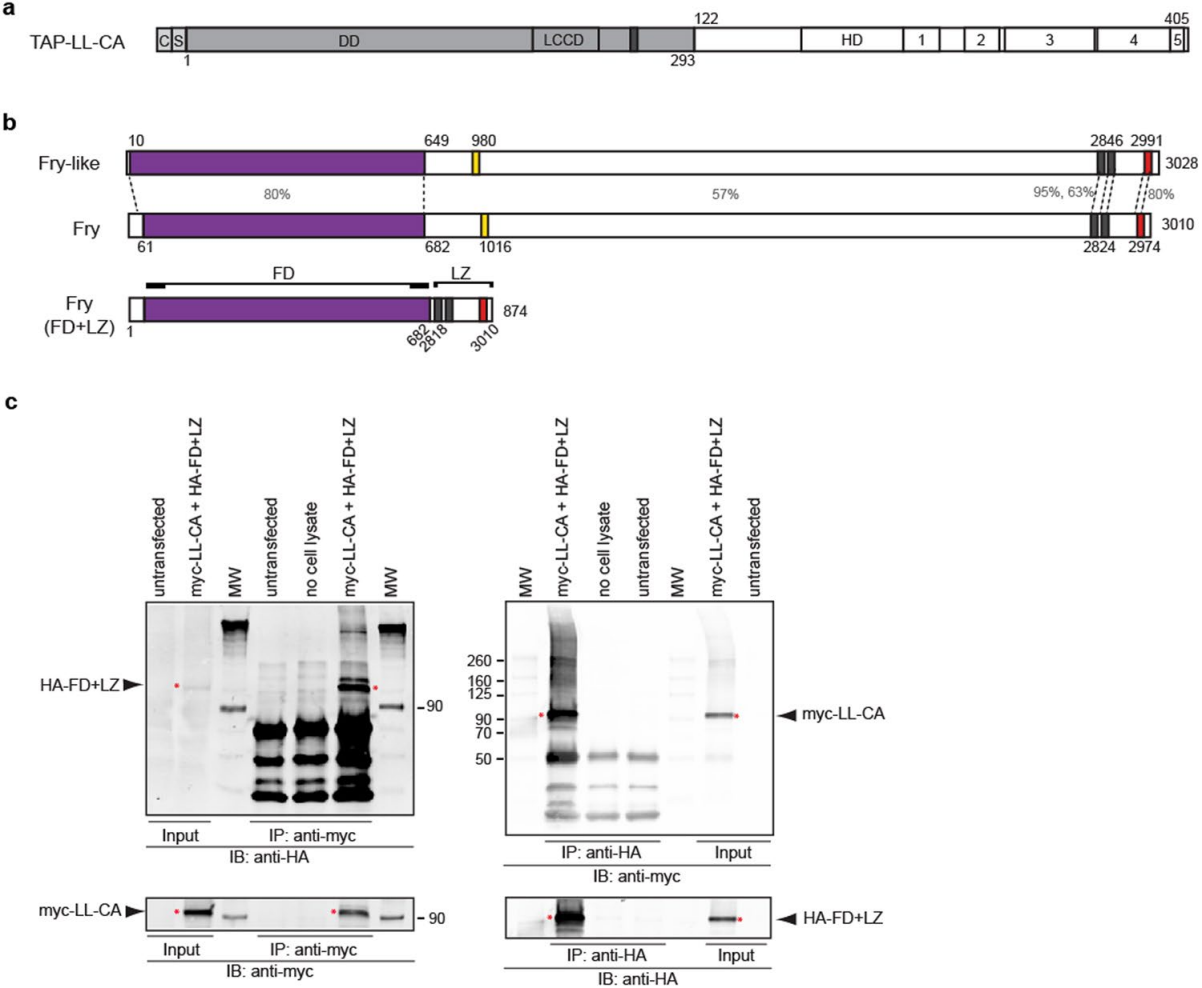
The functional domains of Fry directly interact with constitutive active Ldb1-Lhx1. (**a**) TAP-LL-CA protein contains a calmodulin binding peptide c, a streptavidin binding peptide s, dimerization domain (DD) of Ldb1 protein, Ldb1-Chip conservative domain (LCCD), nuclear localization signal (dark gray bar), Lhx1 homeodomain (HD), and Lhx1 C-terminal conserved regions (1–5). (**b**) Full-length *Xenopus* Fryl and Fry proteins. Furry domain (FD, purple bar), two leucine-zipper motifs (gray bars) and a coiled-coil structure (red bar). The percentage of sequence similarity between different regions of these proteins is indicated. The FD + LZ domain fusion version of Fry contains the N-terminal FD domain and C-terminal domains (LZ). The aminoacid numbers of the original protein are indicated. (**c**) Western blot analysis of immunoprecipitated complexes of transfected HEK-293T cells. Cells were co-transfected with myc-LL-CA and HA-FD + LZ, immunoprecipitated (IP) and blotted (IB) as indicated. IP of untransfected cells and without cell lysate were used as controls for the assay. The red asterisks indicate the bands of interest.

To identify proteins associated with the Ldb1-Lhx1 complex, we purified proteins that interact with a constitutively-active form of Lhx1 (LL-CA)^11,45^ (Fig. 1a). We transiently expressed TAP-LL-CA in renal A6 epithelial kidney cells, which endogenously express *lhx1*^46,47^. Following TAP purification, the samples were analyzed by nano-liquid chromatography coupled with tandem mass spectrometry (nanoLC-MS/MS). A complete list of interacting proteins has been uploaded to the ProteomeXchange Consortium. Using a unique peptide cutoff of two or more, we identified 403 proteins that interacted with the TAP-LL-CA sample. As validation of our TAP purification assay, 13 proteins previously shown to interact with Ldb1-Lhx1 were identified^26^. These are shown in Table 1, along with other interactors that have similar expression patterns to *lhx1* or known roles in the kidney. One protein of interest was Fryl since it is known to interact with Fry in a protein complex^48^, and Fry is known to play a role embryonic axis development, similar to Lhx1^13,35^. We first confirmed the TAP data, by demonstrating myc-LL-CA and endogenous FRYL interact by co-immunoprecipitation in HEK-293T cells (Supplementary Fig. S1).

**Table 1:** Selected proteins identified by TAP purification of TAP-LL-CA followed by nanoLC/MS/MS.

Identified Proteins	Accession Number	Number of unique peptides TAP-LL-CA
LIM-domain-binding protein 1b	BAE95405.2	11
LIM/homeobox protein Lhx1	NP_001084128.1	2
single-stranded DNA binding protein 2 S homeolog	NP_001080347.1	4
annexin A2 S homeolog	P27006	27
LIM domain and actin binding 1 L homeolog	NP_001088398	19
ARP3 actin-related protein 3 homolog	AAH64225	12
eukaryotic translation elongation factor 2 L homeolog	NP_001080656	9
serine/threonine-protein phosphatase PP1-beta catalitic subunit	NP_001085426	6
similar to t-complex polypeptide 1	NP_001079566	4
Ras related S homeolog	NP_001085764	3
guanine nucleotide-binding protein subunit beta-1 S homeolog	NP_001084140	9
14-3-3 zeta protein L homeolog	CAA64773	2
DNA replication licensing factor mcm5-A L homeolog	NP_001080893	2
developmentally-regulated GTP-binding protein 1 L homeolog	P43690	2
FRY like transcriptional coactivator	XP_002933492	4
kinesin family member 5B	AAI67608	2
tropomodulin 3 S homeolog	NP_001080242	2
copine I L homeolog	NP_001083652	6
catenin delta-1 L homeolog	NP_001082468	13
stomatin L homeolog	NP_001080162	5
solute carrier family 25 member 3 L homeolog	NP_001080195	2
flightless 1 S homeolog	NP_001086319	4
tyrosine-protein kinase Src-1 S homeolog	NP_001079114	3
ankyrin repeat domain 13A L hoemolog	NP_001088043	3
keratin 19 S homeolog	AAI23172	11
rac1 L homeolog	NP_001089332	13
IQ motif containing GTPase activating protein 2	NP_001082588	30
ras homolog gene family, member A L homeolog	NP_001079729	10
Rho GTPase Cdc42 L homeolog	AAG36944	12
villin like L homeolog	NP_001082488	12
kras S homeolog	NP_001081316	11
myristoylated alanine rich protein kinase C substrate S homeolog	NP_001080075	5
ras GTPase-activating-like protein IQGAP1	XP_002932312	4
Guanine nucleotide-binding protein G(s) subunit alpha S homeolog	P24799	3
ras GTPase-activating-like protein IQGAP2	XP_002934891	3
calpain 5 L homeolog	NP_001080808	3
CYcloPhilin	NP_001080260	3

List of proteins identified in *Xenopus* kidney A6 cells and previously reported as Lhx1 interactors, known to be expressed, have a function in the kidney and/or the organizer. The number of unique identified peptides are indicated for each protein. Full experimental protein list was submitted to the ProteomeXchange Consortium with the identifier PXD006926.

Since Fryl FD and LZ domains are also present in the related protein Fry (Fig. 1b) and rather than using the full-length Fry protein, which is 330 kDa in size, we utilized the FD + LZ (~100 kDa) (Fig. 1b)^35^, and test interaction between Lhx1 and Fry functional domains. We confirmed the interaction of HA-FD + LZ and myc-LL-CA by reciprocal paraformaldehyde crosslinked co-immunoprecipitation in HEK-293T cells (Fig. 1c). We performed GST pull-down assays to verify the interaction between FD + LZ and GST-LL-CA, as well as with a truncated version lacking the C-terminal domains of Lhx1 (Supplementary Fig. S2). Together, our results demonstrate Ldb1-Lhx1 can complex not only with Fryl, but also with the FD + LZ domains of Fry.

### Fry is expressed in the kidney field

**Figure 2:**
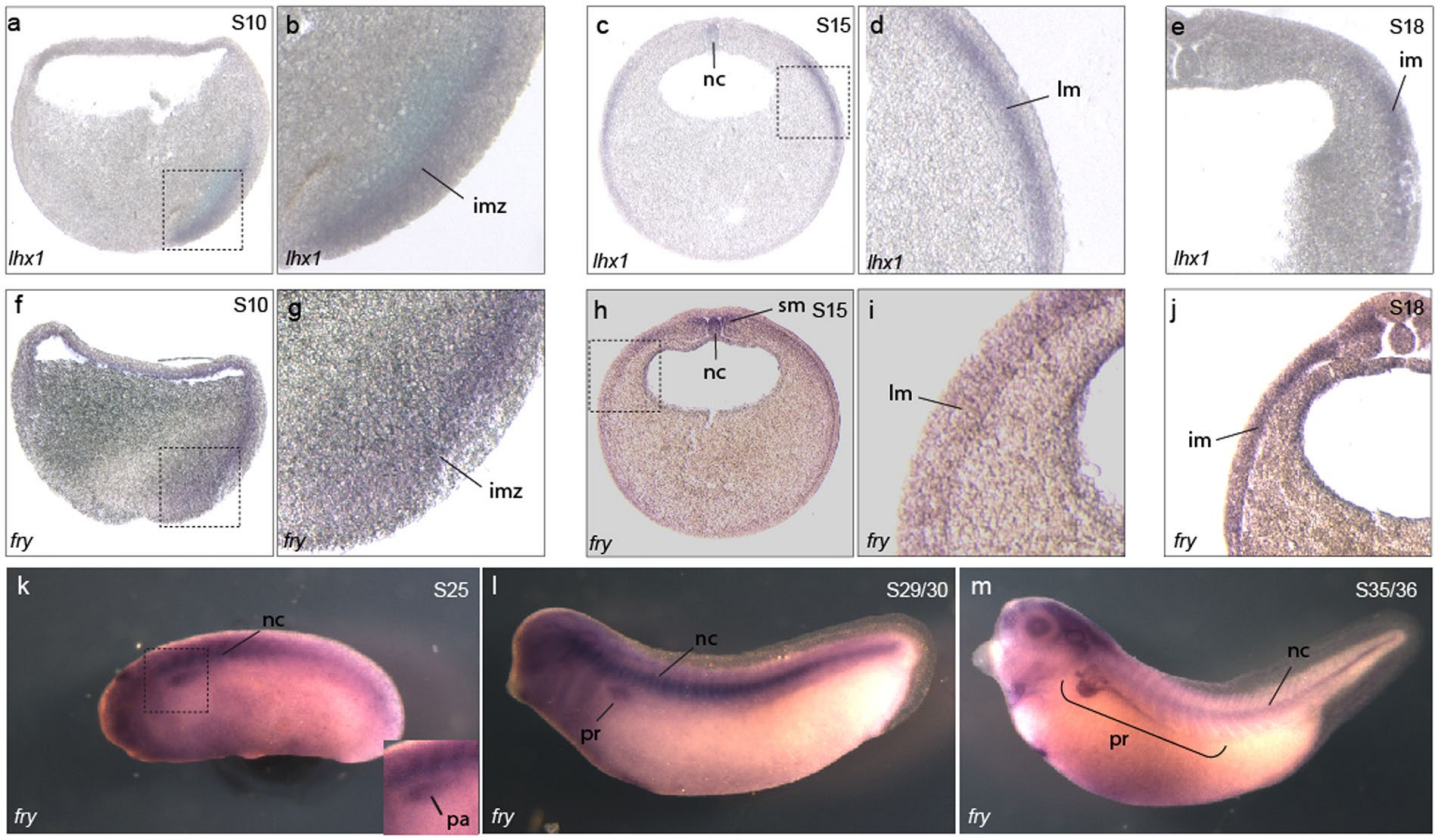
*Fry* is co-expressed with *lhx1* in the intermediate mesoderm and pronephric kidney of *Xenopus* embryos. (**a**–**m**) *Fry* and *lhx1* expression in *Xenopus* embryos. (**a**) Crossed section of a S10 embryo stained for *lhx1*. (**b**) Magnification of the region marked in a. (**c**,**d**) Transverse section of a S15 embryo with *lhx1*. (**d**) Magnification of the area marked in c. (**e**) Expression of *lhx1* in a S18 embryo. (**f**) Crossed section of a S10 embryo stained for *fry*. (**g**) Magnification of the area marked in f. (**h**,**i**) Expression of *fry* in S15 embryos. (**h**) Transverse section. (**i**) Magnification of the marked area in h. (**j**) Expression of *fry* in a S18 embryo. (**k**–**m**) Expression of *fry* in tadpole stages. Involuting marginal zone (imz), lateral mesoderm (lm), pronephric anlage (pa), intermediate mesoderm (im), notochord (nc), somitic mesoderm (sm), pronephros (pr). Representative embryos are shown.

To determine if either gene is expressed in similar tissues to *lhx1*, we performed *in situ* hybridization to characterize *fry* and *fryl* mRNA expression. In *Xenopus* embryos *fry* is expressed in the involuting mesoderm of early gastrula overlapping with *lhx1*, while *fryl* expression is not detected in this region (Fig. 2a,b,f,g and Supplementary Fig. S3). At the beginning of neurulation, *fry* and *fryl* are detected in the presomitic mesoderm and notochord (Fig. 2h,i and Supplementary Fig. S3) and *fry* is additionally expressed in a region of the lateral plate mesoderm (LPM) and IM where *lhx1* is also present (Fig. 2c–e,h–j). In tailbud stages, both *fry* and *fryl* are similarly expressed in the somites and notochord (Fig. 2k–m and Supplementary Fig. S3). At this time in development, *fry* is additionally expressed in the kidney anlage (Fig. 2k), and later becomes restricted to the nephrostomes, proximal and early distal tubules (Fig. 2l,m). Thus, *fry* exhibits a broader area of expression and overlaps with *lhx1* in the kidney. Because both Fry and Lhx1 have known roles in mesoderm patterning and axis development and are expressed during pronephros formation, we reasoned that this interaction might be required for kidney development.

### Fry is required for pronephros development

**Figure 3:**
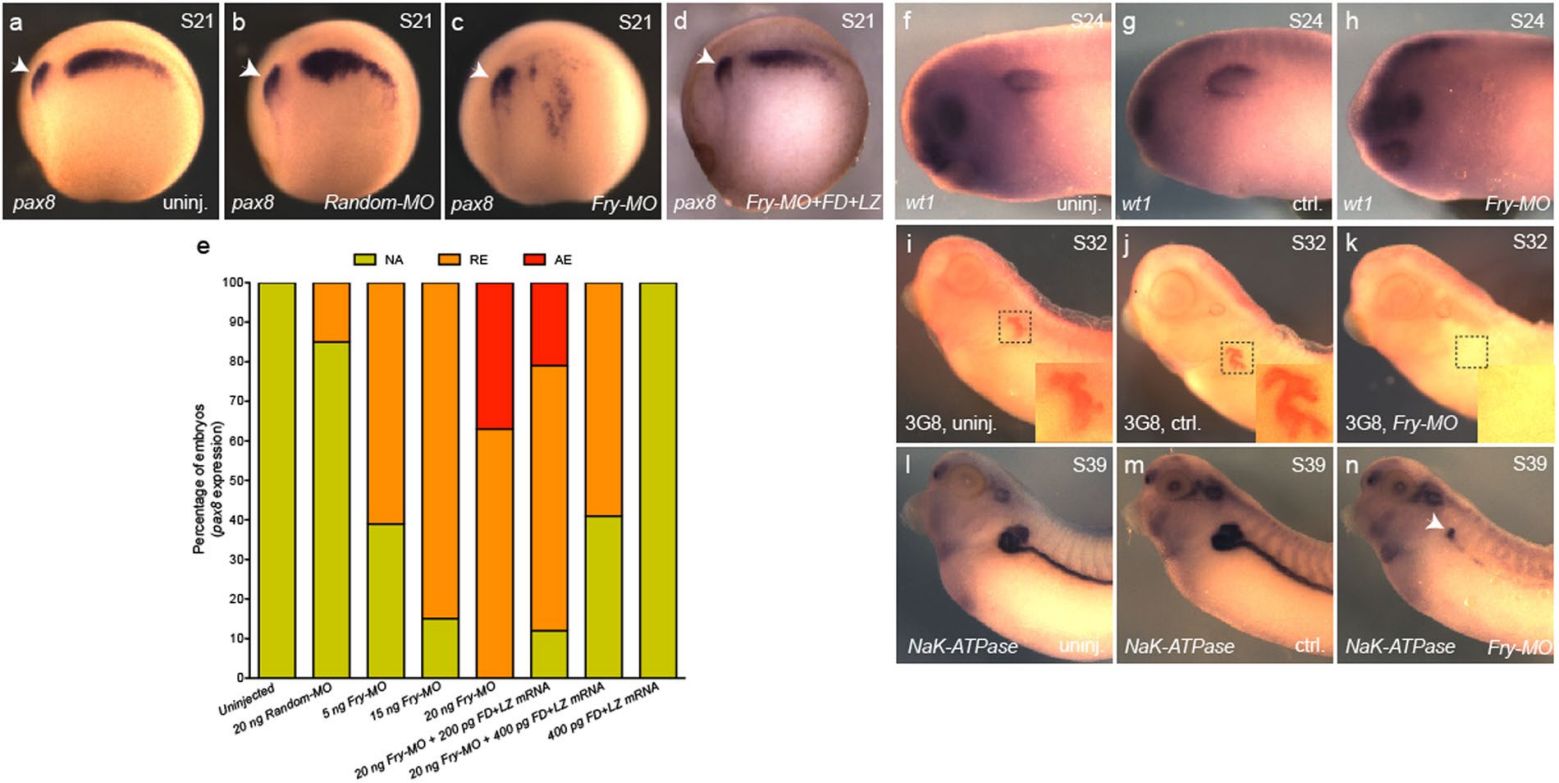
The size of the kidney field is reduced in Fry*-*depleted embryos. (**a**–**d**) *Pax8* expression in S21 (stage 21) embryos, lateral views. Arrow points to the otic vesicle. (**a**) Uninjected embryo. (**b**) *Random-MO* injected embryo. (**c**) *Fry-MO* injected embryo. (**d**) *Fry-MO* + FD + LZ mRNA (400 pg) coinjected embryo. (**e**) Percentage of embryos with reduced (RE), absent (AE) or not affected (NA) *pax8* expression. Uninjected (N = 2, 35), *Random-MO* (N = 2, 23), *Fry-MO* 5 ng (N = 2, 18), 15 ng (N = 3, 53), 20 ng (N = 4, 70), *Fry-MO* 20 ng + FD + LZ mRNA 200 pg (N = 2, 39), *Fry-MO* 20 ng + FD + LZ mRNA 400 pg (N = 2, 51), FD + LZ mRNA 400 pg (N = 2, 46). Data on graph is presented as mean. (**f**–**h**) *wt1* expression in S24 (stage 24) embryos. (**f**) Uninjected (*n* = 20), (**g**) control and (**h**) injected (*n* = 20) sides of the same embryo are shown. (**i**–**k**) 3G8 immunostaining. (**i**) Uninjected (*n* = 25), (**j**) control and (**k**) injected (*n* = 28) sides of the same embryo. Magnifications of the boxed areas are shown in each panel. (**l**–**n**) *β1-NaK-ATPase* expression in S39 (stage 39) embryos. (**l**) Uninjected (*n* = 22). (**m**) Control and (**n**) injected (*n* = 23) sides of the same embryo, arrow points to pronephros positive staining. Representative embryos are shown. Embryos at 8-cell were injected 1x V2 with 15 or 20 ng of the morpholino or as indicated.

It has been shown by antisense oligonucleotides and transient CRISPR knockdown that depletion of *lhx1* results in a reduction in size of the pronephric kidney^11,17^. If Fry plays a role during kidney organogenesis, we would expect Fry depletion to affect the kidney formation. To test this, we depleted *Xenopus fry* transcripts using a validated translation blocking morpholino oligonucleotide (*Fry-MO*), which has been shown to reduce *Xfry-GFP* levels and could be rescued by *Xfry* or *Xfry*-enR + LZ mRNA injections^35^. We injected 8-cell embryos in one V2 blastomere (1 × V2) with *Fry-MO*, to target one of the two kidney fields and minimized the occurrence of gastrulation defects induced by depletion in dorsal blastomeres^35^. We found that *pax8* expression was reduced in *Fry-MO* injected embryos in a dosage dependent manner (Fig. 3). Based upon the range of *Fry-MO* used previously^35^, we injected 20 ng of *Fry-MO*, which resulted in ~60% of the embryos with reduced *pax8* expression and 39% with no *pax8* expression in the pronephric anlage when comparing the injected to the uninjected side of the same embryo (Fig. 3c,e). Injection of 20 ng of *Random-MO* had little or no effect on *pax8* expression (Fig. 3a,b and e). The specificity of the phenotype was verified by co-injecting *Fry-MO* and FD + LZ mRNA (not targeted by the morpholino). This resulted in a rescue of *pax8* expression in the kidney anlage (Fig. 3d,e). Additionally, we analyzed the expression of Wilms’ tumor 1 (*wt1*) in the splanchnic layer of the LPM that will form the glomus^49^. We observed reduction or absence of *wt1* expression in 95% of *Fry-MO* injected embryos (Fig. 3f–h). Lastly, to evaluate the definitive kidney, we assessed 3G8 staining of the nephrostomes and proximal tubule and *β1-NaK-ATPase* expression in the tubules and duct. For both, we observed a reduced staining in more than 80% of the *Fry-MO* injected embryos while control embryos were unaffected (Fig. 3i–k,l–n). The reduced expression of pronephric mesenchyme markers after Fry depletion demonstrates that Fry is required to specify the pronephric progenitors.

### Fry depletion in the kidney field alters expression of paraxial mesoderm genes

**Figure 4:**
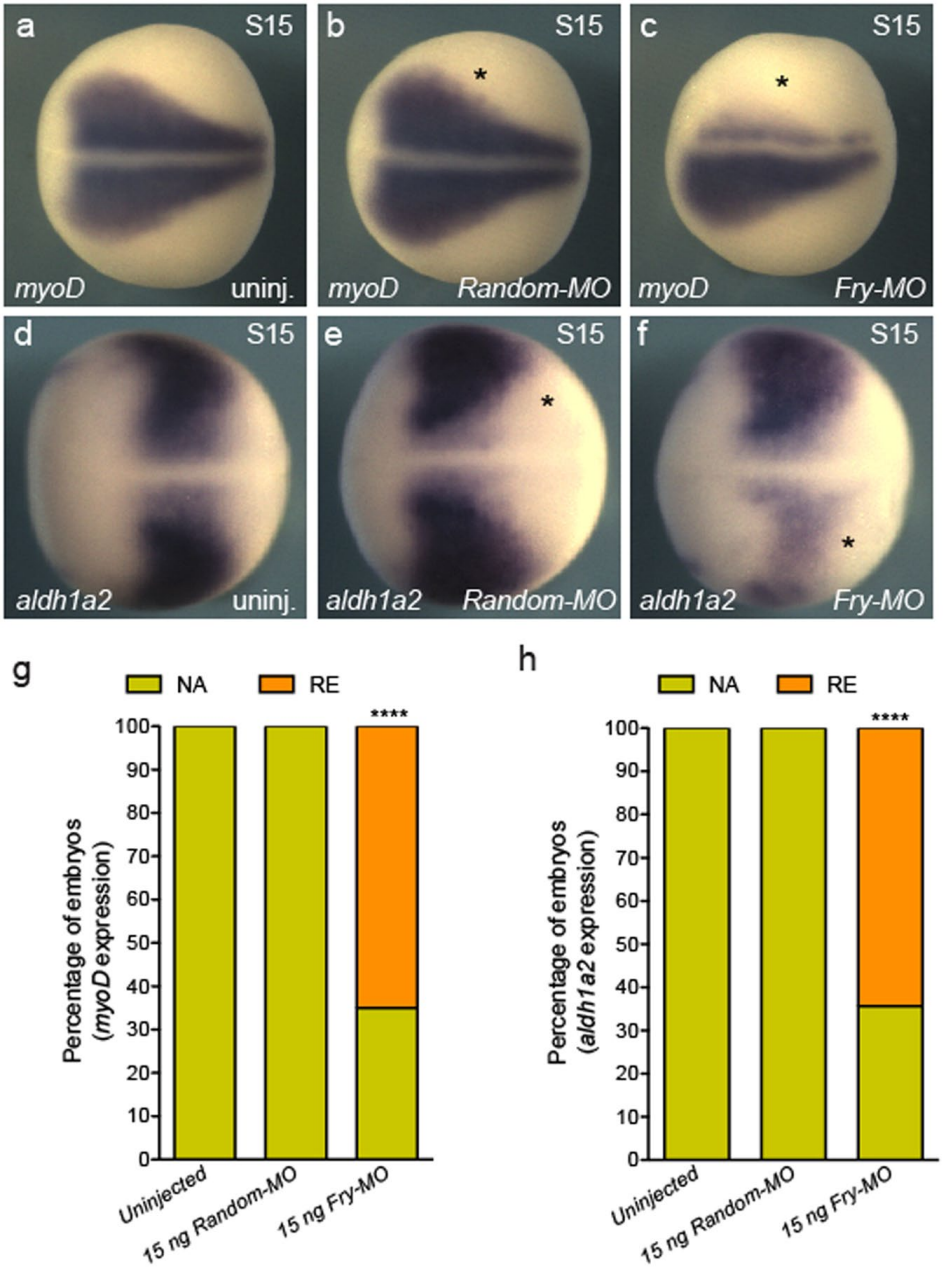
Expression of paraxial mesoderm marker genes is reduced in Fry-depleted embryos. (**a**–**c**) *In situ* hybridization of S15 (stage 15) embryos for *myoD*. (**g**) Percentage of embryos with reduced (RE) or not affected (NA) *myoD* expression field revealed by *in situ* hybridization. Uninjected (N = 3, 92), *Random-MO* (N = 2, 36), *Fry-MO* (N = 4, 85). (**d**–**f**) *In situ* hybridization of S15 embryos for *aldh1a2*. (**h**) Percentage of embryos with reduced (RE) or not affected (NA) *aldh1a2* expression field revealed by *in situ* hybridization. Uninjected (N = 3, 75), *Random-MO* (N = 2, 36), *Fry-MO* (N = 3, 76) (N = number of experiments, number of embryos). As indicated on each panel, 8-cell embryos were injected 1x V2 with 15 ng of the *MOs* or left uninjected. Asterisk indicates the injected side of the embryo. Representative embryos are shown. Data in graphs is presented as means. Statistical significance was evaluated using Fisher’s exact test ^***^p < 0.0001.

The formation of the pronephric anlage from the IM requires inductive signals emanating from the PM^11,50–52^. Since *Fry* is expressed throughout the trunk mesoderm (Fig. 2h–m), we evaluated the consequences of *fry* depletion in the V2 blastomere for expression of PM markers, using 15 ng of *Fry-MO* a dose that reduces the size of the kidney field without entirely loosing expression of *pax8,* as seen with 20 ng of *Fry-MO* (Fig. 1c,e). We assayed for *myoD* expression, one of the earliest muscle-specific response factors^53^. Injection of *Fry-MO* (15 ng) resulted in reduced *myoD* expression amongst 65% of S15 embryos, whereas *myoD* expression in *Random-MO* or uninjected embryos appeared unaffected (Fig. 4a–c and g). We also evaluated expression of *aldh1a2*, a modulator of RA signaling and necessary for the specification of the kidney field^54^. In *Xenopus* embryos, *aldh1a2* transcripts initially localize to the involuting mesoderm of the gastrula and later become restricted to the PM^55^. We observed a pan-tissue reduction in *aldh1a2* expression in 64% of the embryos injected with *Fry-MO* while *Random-MO* injected and uninjected embryos were unaffected (Fig. 4d–f and h). Thus, the reduction of *myoD and aldh1a2* expression following *fry* depletion in the V2 blastomere indicates that Fry has broad effects on mesoderm patterning.

### Fry and Lhx1 act synergistically in pronephros patterning

**Figure 5:**
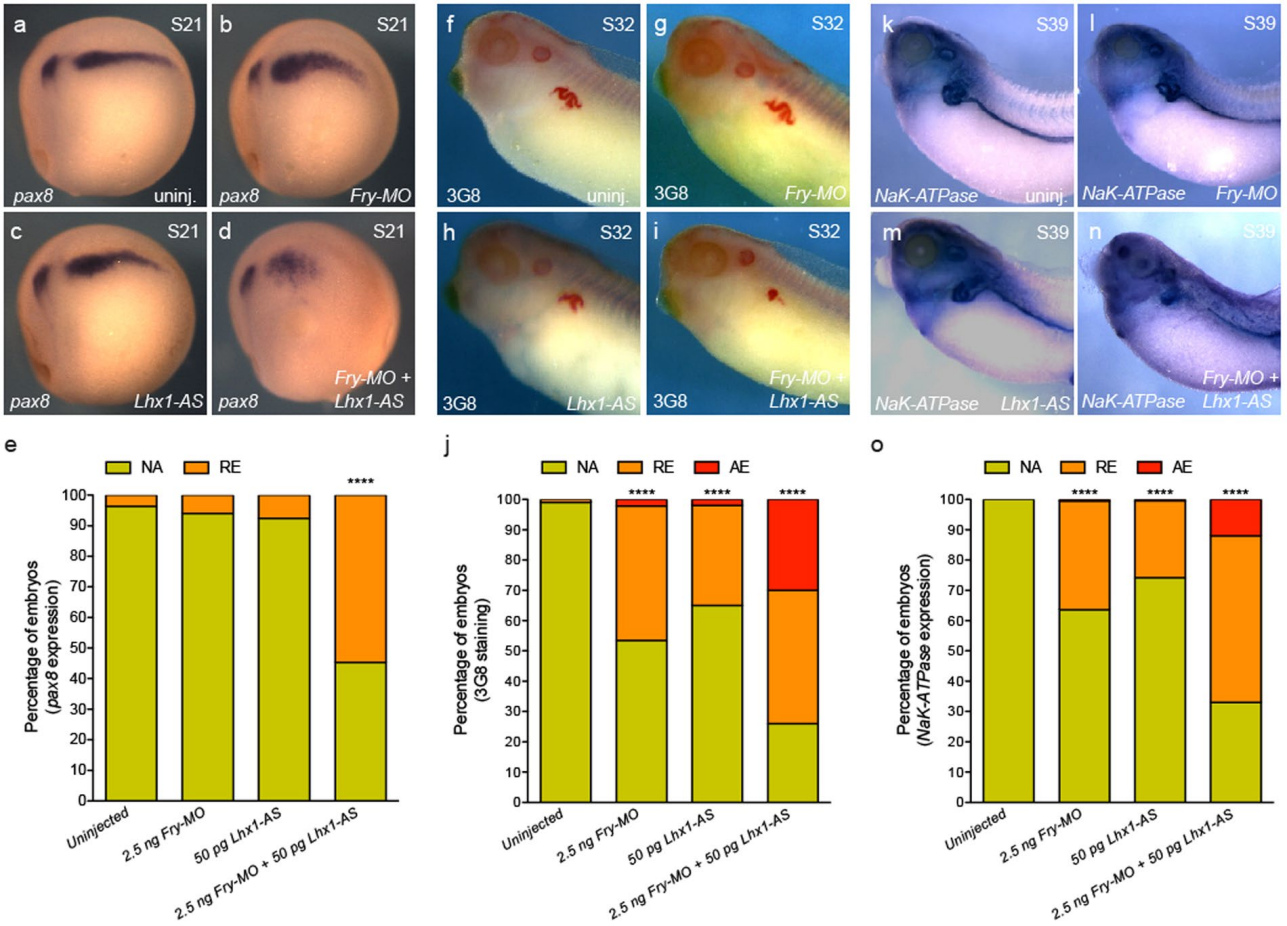
Synergistic interaction of Fry and Lhx1 to pattern the kidney field. (**a**–**d**) *Pax8* expression in S21 (stage 21) embryos, lateral views. (**e**) Percentage of S21 embryos with abnormal *pax8* expression under different treatments. Uninjected (N = 3, 41), *Fry-MO* (N = 3, 56), *Lhx1-AS* (N = 3, 65), *Fry-MO* + *Lhx1-AS* (N = 3, 75) (N = number of experiments, number of embryos). (**f**–**i**) 3G8 immunostaining of S32 (stage 32) embryos. (**j**) Percentage of S32 embryos with reduced or absent 3G8 staining. Uninjected (N = 3, 122), *Fry-MO* (N = 3, 88), *Lhx1-AS* (N = 3, 79), *Fry-MO* + *Lhx1-AS* (N = 3, 81). (**k**–**n**) *β1-NaK-ATPase* expression in S39 (stage 39) embryos. (**o**) Percentage of embryos with abnormal *β1-NaK-ATPase* expression. Uninjected (N = 6, 168), *Fry-MO* (N = 6, 165), *Lhx1-AS* (N = 6, 193), *Fry-MO* + *Lhx1-AS* (N = 6, 183). Reduced (RE), absent (AE) or not affected (NA) expression/staining. 8-cell embryos were injected 1x V2 with 2.5 ng of *Fry-MO* and/or 50 pg of *Lhx1-AS*. Representative embryos are shown. Data in the graphs is presented as means. Statistical significance was evaluated using Fisher’s exact test ^****^p < 0.0001.

Lhx1 and Fry interact and depletion of either has similar effects on kidney development. We next addressed whether Fry and Lhx1 act synergistically to pattern the kidney. We used suboptimal doses of *Fry-MO* and antisense oligonucleotides against *lhx1* (*Lhx1-AS*) that have little effect on kidney marker when injected individually. *Lhx1-AS* is an N,N-diethylethylenediamine antisense oligonucleotide previously validated for targeted degradation of the *lhx1* transcripts, can be rescued by exogenous zebrafish *lhx1a* mRNA resistant to binding the oligo, and phenocopies *lhx1* CRISPR knockdown^11,13,17^. A low percentage of embryos injected with 2.5 ng of *Fry-MO* exhibited reduced *pax8* expression (6%); a similar percentage of embryos showed reduced *pax8* expression by the injection of 50 pg of *Lhx1-AS* (8%) (Fig. 5a–c and e). When co-injected *Fry-MO* and *Lhx1-AS* at these suboptimal doses, we observed 55% of the embryos with reduced *pax8* expression (Fig. 5d,e), thus demonstrating that Fry acts synergistically with Lhx1 in the kidney anlage formation.

To evaluate how Fry and Lhx1 synergism affects pronephric terminal differentiation, we assessed 3G8 staining and *β1-NaK-ATPase* expression. 3G8 immunostaining of the proximal tubule was reduced in 47% of *Fry-MO* injected embryos and 35% of *Lhx1-AS* embryos while 74% of the embryos co-injected with the synergistic dose showed reduced or no staining (Fig. 5f–j). Similar results were observed by *in situ* hybridization for *β1-NaK-ATPase* (Fig. 5k–o). Together, these experiments demonstrate a synergistic effect of Fry and Lhx1 on pronephros patterning ultimately affecting proper pronephric tubule formation.

**Figure 6:**
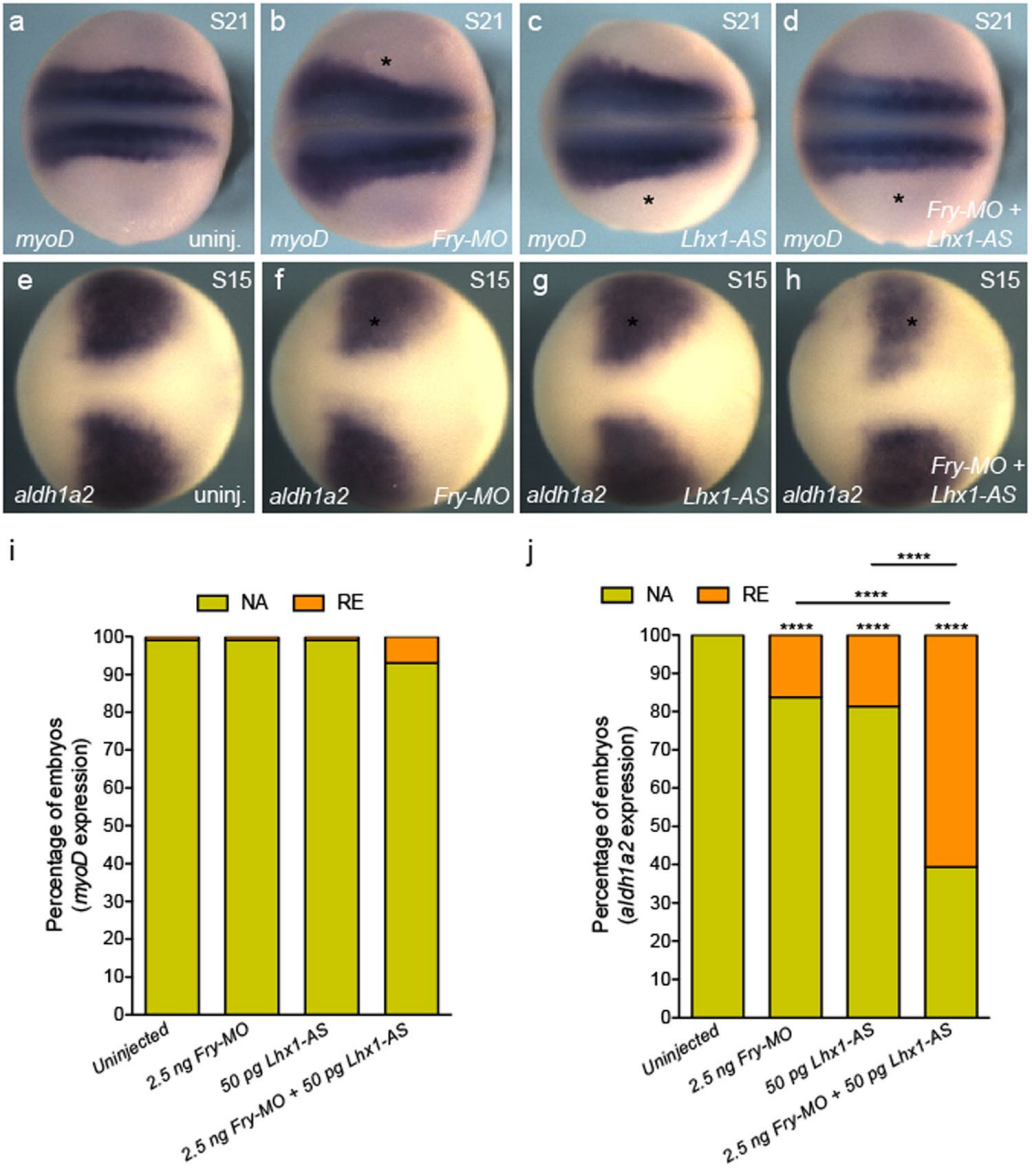
Synergistic interaction of Fry and Lhx1 in the paraxial mesoderm. (**a**–**d**) *In situ* hybridization of S21 (stage 21) embryos for *myoD*. Asterisk indicates the injected side of the embryo. (**e**–**h**) *In situ* hybridization of S15 embryos for *aldh1a2*. (**a**,**e**) Uninjected embryos. (**i**) Percentage of embryo with reduced (RE) or not affected (NA) *myoD* expression. Uninjected (N = 3, 85), *Fry-MO* (N = 3, 84), *Lhx1-AS* (N = 3, 78), *Fry-MO* + *Lhx1-AS* (N = 3, 73). (**j**) Percentage of embryo with reduced (RE) or not affected (NA) *aldh1a2* expression. Uninjected (N = 3, 82), *Fry-MO* (N = 3, 86), *Lhx1-AS* (N = 3, 88), *Fry-MO* + *Lhx1-AS* (N = 3, 84) (N = number of experiments, number of embryos). Embryos at 8-cell were injected 1x V2 with 2.5 ng of *Fry-MO* and/or 50 pg of *Lhx1-AS* as indicated on each panel. Representative embryos are shown. Data in graphs is presented as means. Statistical significance was evaluated using Fisher’s exact test ^****^p < 0.0001.

We next tested if concomitant loss of *fry* and *lhx1* affects other mesoderm derivatives, since expression of PM marker genes is reduced in the absence of Fry (Fig. 4). We co-injected *Fry-MO* and *Lhx1-AS* at the aforementioned suboptimal doses and assessed the expression of *myoD* and *aldh1a2*. Co-injection of *Fry-MO* and *Lhx1-AS* resulted in a reduction of *myoD* expression in a small percentage of embryos (7%), whereas *Fry-MO* or *Lhx1-AS* individually injected embryos appeared unaffected (Fig. 6a–d,i). Co-injection of *Fry-MO* and *Lhx1-AS* resulted in a mild reduction of *aldh1a2* expression in 61% of the embryos, while injection of *Fry-MO* or *Lhx1-AS* had milder effects on *aldh1a2* expression (16% and 19%); however, this was significantly higher than uninjected embryos showing a reciprocity of *aldh1a2* expression between the PM and IM (Fig. 6e–h,j). These results demonstrate the specificity of the synergistic effect of Fry and Lhx1 in the IM where both are expressed.

### Lhx1 and Fry regulate miRNA levels

**Figure 7:**
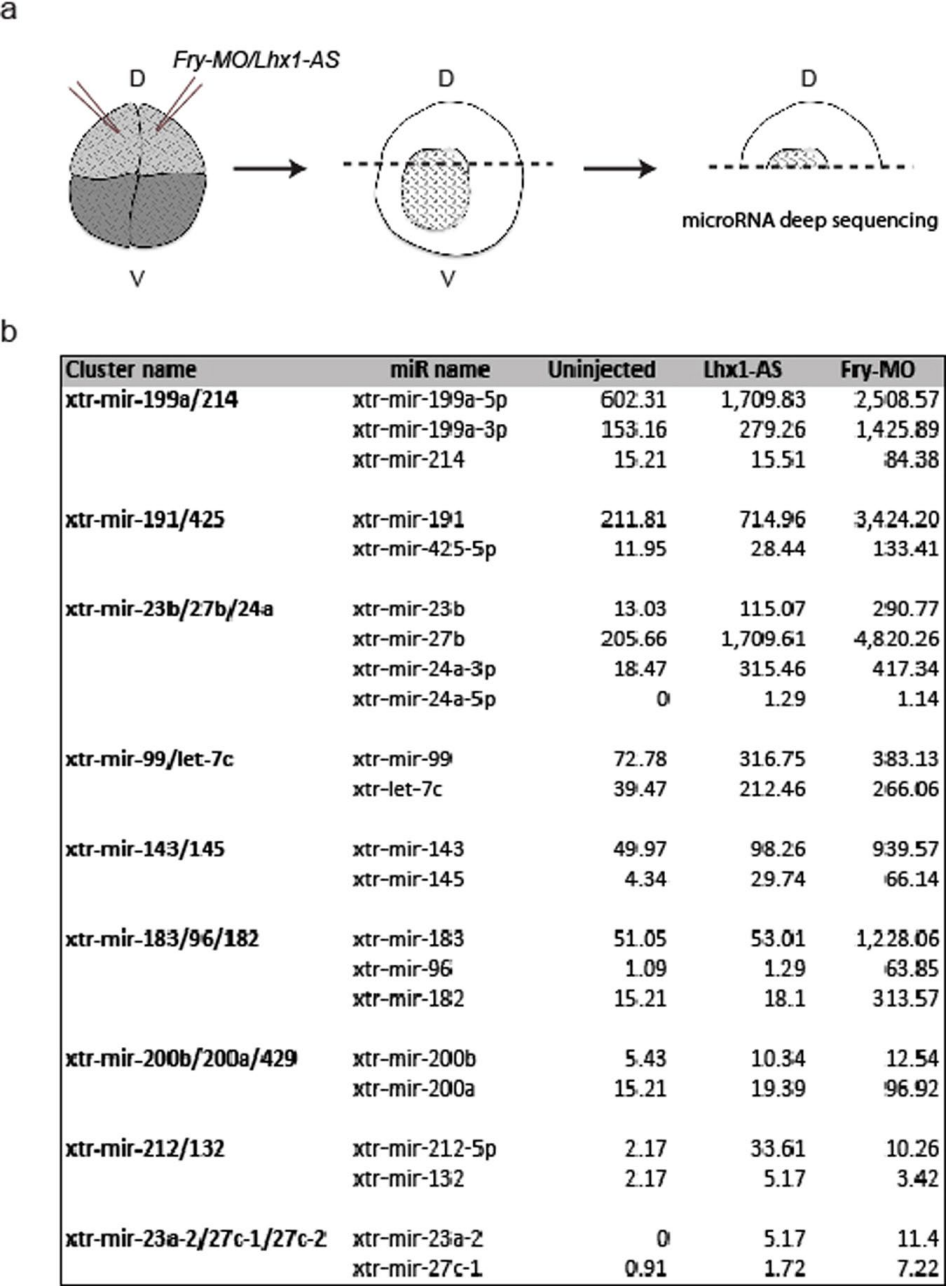
miRNA deep sequencing of Fry- and Lhx1*-*depleted embryos. (**a**) Experimental procedure followed to generate the samples for miRNA deep sequencing. Both dorsal blastomeres of 4-cell embryos were injected. Dorsal halves were isolated and processed for miRNA deep sequencing. (**b**) Selected nine miRNA clusters with increased levels of their respective miRs upon either *lhx1* or *fry* depletion. The values are the normalized number of counts for each miR.

In *Xenopus*, Fry is known to regulate miRNA expression^35^. As we have found that Lhx1 and Fry coordinate activity in kidney formation, we evaluated if these proteins regulate miRNAs in common during this process. To identify potential miRNA targets, we performed miRNA deep sequencing of embryos depleted of either *lhx1* or *fry*. In addition to the kidney, *lhx1* and *fry* are co-expressed in the dorsal mesoderm of *Xenopus* where both function in axial mesoderm (AM) patterning. We chose to perform our screen using organizer tissue for multiple reasons: the organizer requires Fry and Lhx1 activity^13,15,35^, the tissue is easy to isolate unlike pronephric kidney, and miRNAs are often widely expressed. We selected doses of *Fry-MO* (20 ng) and *Lhx1-AS* (400 pg) that induce a shortened axis and headless phenotype at S33/34 by injecting both dorsal cells of 4-cell embryos (2x D) (Supplementary Fig. S4). We performed microRNA deep sequencing on isolated dorsal tissue from early gastrula stage embryos that were dorsally-depleted of either *fry* or *lhx1* (Fig. 7a). We identified a total of 1,987 unique, known and predicted, miRNAs of different chordata species, 257 of which correspond to *Xenopus*, 28 specific to *Xenopus laevis* and 229 to *Xenopus tropicalis* and new to *X. laevis*. miRNAs are often found in clusters that are co-transcribed as a single pri-miRNAs and later processed into individual pre-miRNAs^56,57^. This genomic co-localization could result in joint transcriptional regulation by a common transcriptional complex^58^ and for this reason, we focused on known miRNAs which lie within genomic clusters^59^. This yielded a total of 178 unique miRNAs of different chordata species constituting 98 clusters with 2 or more detected members, out of which 27 correspond to known *Xenopus* miRNAs clusters.

**Figure 8:**
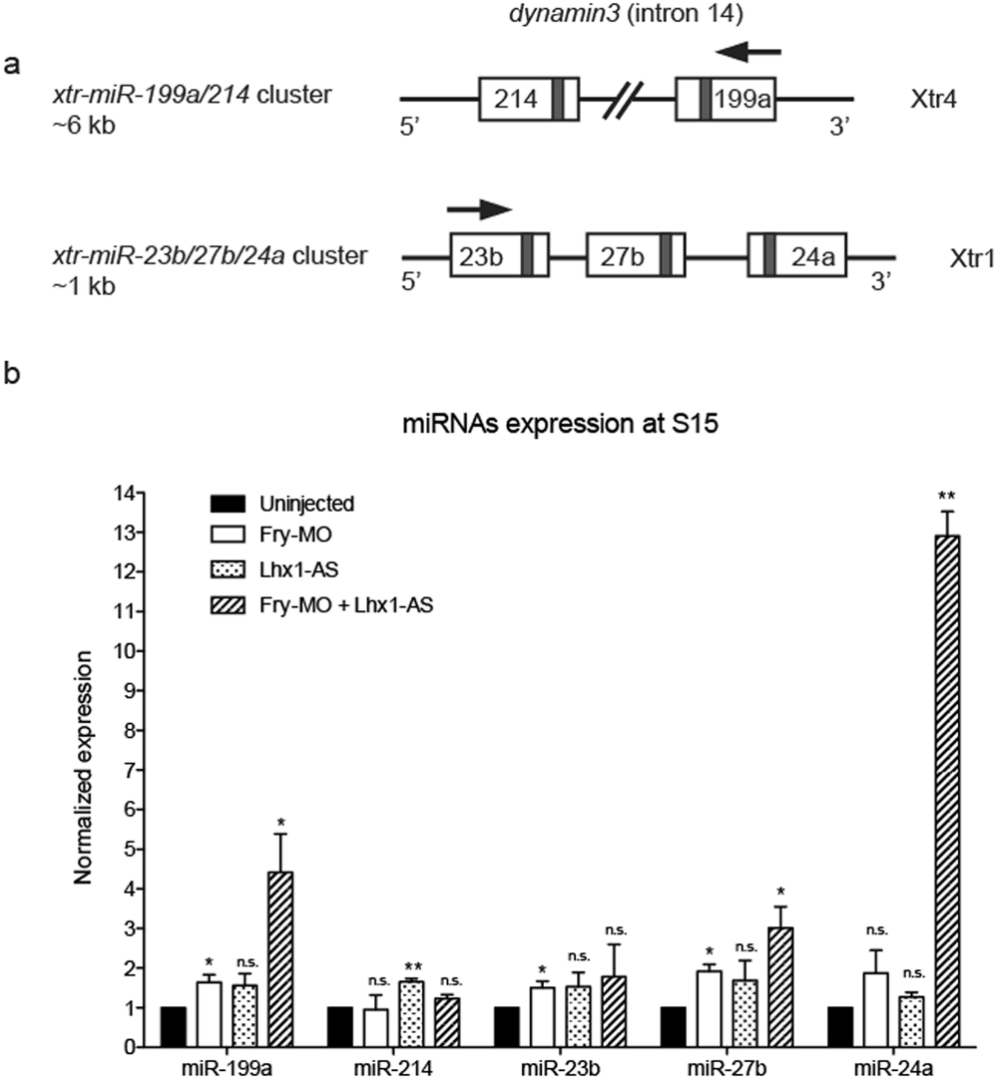
Validation of miRNA deep sequencing data by real-time qRT-PCR. (**a**) Schematic of the miR-199a/214 and miR-23b/27b/24a clusters in the *X*. *tropicalis* genome. The *Xenopus tropicalis* chromosome (Xtr) where each cluster is located is indicated. The miR-199a/214 cluster is located within intron 14 of the dynamin3 gene and has a length of ~6 kb while the miR-23b/27b/24a cluster is intergenic and has a length of ~1 kb. The grey colored rectangles indicate the position of the mature miRNAs within the pre-miRNA structures. Arrows indicate transcription direction. (**b**) Expression levels of miRs within the miR-199a/214 and miR-23b/27b/24a clusters were determined in Lhx1- and/or Fry-depleted embryos. 8-cell embryos were injected 2x V2 with 2.5 ng of *Fry-MO* and/or 50 pg of *Lhx1-AS*. Error bars indicate standard deviation derived from three repeats of the PCR reactions with different biological samples. Statistical significant differences were determined by a one-tailed paired t-test. n.s., non-significant; ^*^p < 0.05; ^**^p < 0.01.

Considering that Fry was reported to function as a miRNA repressor, we focused on 9 genomic clusters where the levels of all miRNAs members increase upon loss of Fry and Lhx1 (Fig. 7b). These miRNAs clusters are located in chromosomes 1, 2, 3, 4 and 7 and positioned within introns of host protein-coding genes or intergenic regions, as is the case of most *Xenopus* miRNAs^57^. Given that we are interested on the regulation of miRNA by Lhx1 and Fry in kidney development, we focused on two clusters, miR-199a/214 and miR-23b/27b/24a, which are involved in development and disease of this tissue^43,60–63^. Additionally, these two miRNAs clusters are located in different chromosomes and genomic regions with respect to the closest protein-coding gene (Fig. 8a).

To validate the miRNA deep sequencing results with respect to the kidney, we analyzed the expression levels of mature miRNAs within these two clusters during pronephros specification by real-time qPCR in Fry and Lhx1 depleted embryos. We found an increase of miR-199a, miR-27b, and miR-24a in *Fry-MO* and *Lhx1-AS* co-injected embryos. Additionally, miR-214 and miR-23b significantly increased in *Lhx1-AS* and *Fry-MO* injected embryos, respectively (Fig. 8b). Thus, the coordinated activity of Lhx1 and Fry is required to regulate the levels of miR-199a/214 and miR-23b/27b/24a.

**Figure 9:**
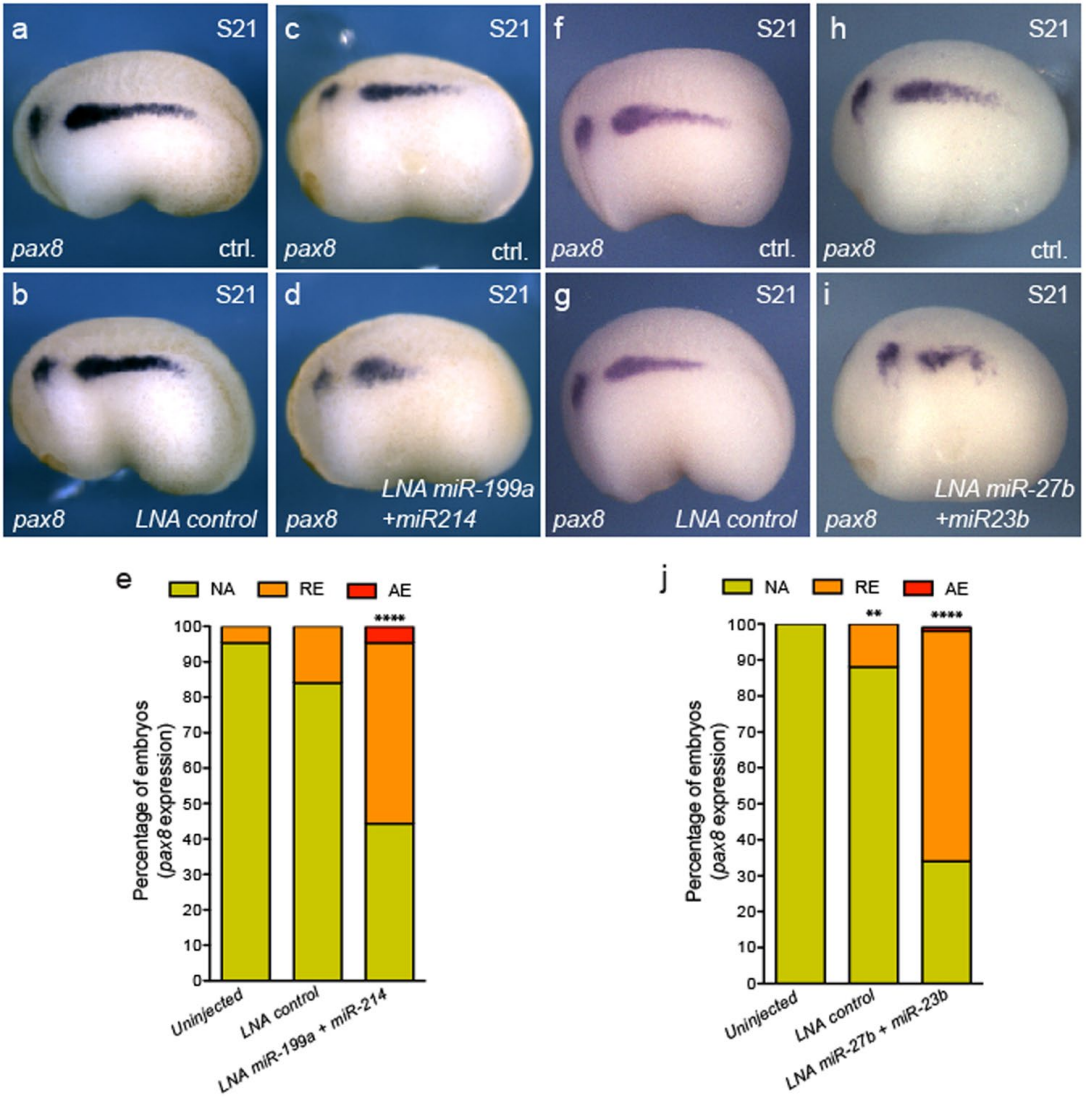
Overexpression of miR-199a/214 and miR-23b/27b reduce the kidney field size. (**a**–**i**) *Pax8* expression in S21 (stage 21) embryos injected with LNA mimics. (**a**,**b**,**f** and **g**) Injected with LNA control. (**a**,**f**) Uninjected side (ctrl). (**b**,**g**) Injected side with LNA control. (**c**,**d**) Embryo injected with LNA miR-199a + miR214. Uninjected (**c**) and injected (**d**) sides of the same embryo. (**h**,**i**) Embryos injected with LNA miR-27b + miR23b. Uninjected (**h**) and injected (**i**) sides of the same embryo. (**e**) Percentage of embryos with abnormal *pax8* expression field. Uninjected (N = 3, 63); LNA ctrl. (N = 3, 64); LNA miR-199a + miR-214 (N = 3, 76, 49% reduced or absent expression) (N = number of experiments, number of embryos, % of affected embryos). (**j**) Percentage of embryos with abnormal *pax8* expression field. Uninjected (N = 3, 66); LNA ctrl. (N = 3, 65); LNA miR-27b + miR-23b (N = 3, 59, 64% reduced or absent expression). Reduced (RE), absent (AE) or not affected (NA) field of expression. Data in graphs is presented as means. ^****^p < 0.0001, ^**^p < 0.01 Fisher’s exact test.

We next determined whether the miR-199a/214 and miR-23b/27b/24a clusters play a functional role in the kidney. If Lhx1 and Fry are required to repress miRNAs for kidney tissue specification to occur, it would stand to reason that perturbance of individual miRNA clusters in the developing kidney should result in a reduction of kidney markers. To test this, we overexpressed miR-199a/214 or miR-23b/27b miRNAs by injecting the synthetic LNA mimics into one V2 blastomere of 8-cell embryos and evaluated expression of pronephric kidney genes (Fig. 9 and Supplementary Fig. S5). Currently, commercial pre-designed LNAs to *Xenopus* miR-24a are not available from Exiqon and therefore was not tested in this study. The expression domains of *pax8* and *β1-NaK-ATPase* were significantly reduced in embryos injected with LNA miR-199a and miR-214 mimics (Fig. 9a-e and Fig. S5a–e) as well as with LNA miR-23b and miR-27b mimics (Fig. 9f–j and Supplementary Fig. S5). As expected, V2 targeted injection of LNA miR-199a and miR-214 mimics or LNA miR-23b and miR-27b mimics had insignificant effects on *myoD* or *aldh1a2* expression (Supplementary Fig. S6). Together, these results demonstrate that these microRNA clusters have antagonist effects on kidney specification and their levels are regulated by Lhx1 and Fry.

## Discussion

Lhx1 is required for the specification of the pronephric kidney^11^ and forms complexes with Ldb1 and other cofactors to facilitate the expression of target genes^12,22,23,25,26,64^. However, in the IM, it remains unclear what proteins interact with Lhx1 to specify renal progenitors. In our TAP assays, known components of the core complex were isolated (Lhx1, Ldb1b, Ssbp2); however, other cofactors such as Otx2 and Foxa2, known to interact with Lhx1 in axis and head development^26^, were not identified. This suggests tissue-specific cofactors likely modulate the function of the Lhx1 core complex. We demonstrate that Fryl and Fry bind to the Ldb1-Lhx1 complex. The C-terminal leucine zipper/coiled coil domain (LZ) and Furry domain (FD) are thought to facilitate Fry’s nuclear localization and transcriptional activity in *Xenopus*^35^ and mediate Fry’s cytoplasmic functions in human cells^30–32,34^. Our *in vivo* and *in vitro* binding data suggests that the region of Fry that interacts with Ldb1-Lhx1 resides within these two domains.

In *Xenopus, fry* expression has been reported in the dorsal mesoderm of gastrula-staged embryos and later in the notochord^35^. Complementary to this, we found that *fry* is also expressed in PM, LPM, and IM. Similar to Lhx1*-*depleted embryos^11^, depletion of Fry within the V2 blastomere results in reduced expression of nephrogenic factors. Additionally, depletion of Fry causes reduced expression of *myoD* and *aldh1a2* suggesting that Fry has a general role in mesoderm patterning. As Fry shows broad mesoderm expression, Fry likely interacts with different proteins to pattern specific mesoderm derivatives. We demonstrated coordinated activity of Lhx1 and Fry in the kidney field using validated antisense and morpholino oligonucleotides for *lhx1* and *fry,* respectively. These oligonucleotides have been shown to reduce either the target mRNA or protein, rescued by synthetic mRNA injections, and show a synergistic activity at low doses validating the specificity of the phenotype. Additionally, by performing our expression analysis early in embryo development (neurula and early tailbud stages), we minimize the induction of side effects such as those associated with a morpholino oligonucleotide-induced immune response^65^.

We hypothesize that Lhx1 and Fry coordinate activity to facilitate kidney development by repressing miRNA expression, indirectly facilitating the expression of inductive factors, such as Pax2 and Pax8, to specify the kidney. In support of this, Lhx1 is initially expressed in the IM in Pax2/Pax8 double-knockout mice, but these animals do not specify the nephric lineage^16^. Instead, while the initial IM forms, definitive kidney genes fail to express and, consequently the IM cells undergo apoptosis. Complimentary to this, Carroll *et al*. demonstrated that Lhx1 over-expression in combination with Pax8 (and to a lesser extent, Pax2) causes expansion of the kidney field with ectopic expression of kidney genes within somitic regions, and concomitant reduction of somitic genes^8^. Based upon this data and our work here, Lhx1 appears to function as a competence factor with Fry to prime the IM, while Pax8 and Pax2 work in concert, likely with additional factors, to specify the kidney^16^. While additional experiments will reveal the mechanistic details on miRNA regulation in the kidney, we demonstrate here for the first time a role of the Ldb1-Lhx1 complex in the regulation of miRNAs expression.

Our deep sequencing experiment identified a large group of miRNAs up-regulated exclusively in the absence of Fry suggesting that this protein also regulates miRNA expression independent of Lhx1. Therefore, we speculate that distinct Fry-complexes may control expression of miRNAs in axial mesoderm, PM, and LPM in the absence of Lhx1 to assist in the expression of cell fate determinants. Such regulation would allow for proper establishment of tissue boundaries in the trunk mesoderm and provide a mechanism for how miRNA expression is controlled in different tissues. An interesting finding within the “Fry regulated” group of miRNAs is miR-30a, which has been shown to bind the 3′UTR of *lhx1* and regulate *lhx1* transcript stability^43^. This result suggests the existence of an additional regulatory step of Ldb1-Lhx1 activity, by positively reinforcing Lhx1 expression through its binding partner Fry.

We found that miR-199a/214 and miR-23b/27b/24a clusters regulate early kidney development, and the phenotypes produced by their over-expression resemble animals depleted for *lhx1* and *fry*. Previous data suggest specification of the pronephric field was miRNA independent, with miRNAs thought to be primarily associated with differentiation and kidney homeostasis^43,44^. We show here that repression of miRNAs in the kidney field is required for specification. Thus, we postulate that, in the IM, Fry coordinates activity with Lhx1 to drive kidney development through miRNA regulation.

Further studies investigating individual miRNA contributions from the identified clusters are needed to determine the “miRNA code” regulating the induction of IM to kidney tissue. While there are many potential targets for each miRNA (miRBase), *bona fide* targets of kidney-expressed genes have not been validated. In vertebrates, the miR-199/214 cluster is located within an intron of the dynamin3 gene and transcribed as a single pri-miR199/214 suggesting common transcriptional regulation of both miRs^66^. This evolutionary conserved cluster has been documented to modulate cell fate and antagonize cellular proliferation during early development^67,68^. Similarly, the miR-23b/27b/24a cluster has been implicated in the regulation of cell proliferation during development and disease^69^. Repression of the miR-23b/27b/24a cluster in the liver promotes bile duct specification^70^. Recently, miR-27 was shown to regulate *Hox* genes expression in order for correct spinal cord boundary formation to occur^38^. Therefore, repression of the miRNAs studied here may promote proliferation/specification of kidney cells by allowing expression of their predicted targets, such as *osr1*, *rara, aldh1a2, ret, pax8*, *hnf1a, and sal1/sal2*. Clearance of these and other miRNAs in this region could provide timing cues for the pronephric kidney field to form. While we point to various possible aspects that remain to be addressed, we have shown here that miRNAs function during specification of the kidney field from the IM earlier than previously anticipated. And lastly, we show that spatial and temporal clearance of a subset of these miRNAs by Lhx1 and Fry are required for proper kidney patterning.

## Methods

See Supplementary Methods for more details.

### Ethics Statement

This study was carried out in strict accordance with the recommendations in the Guide for the Care and Use of Laboratory Animals of the NIH. The protocol (14124978) was approved by the institutional Animal Care and Use Committee of the University of Pittsburgh (Animal Welfare Assurance Number: A3187-01).

### Transfection of Xenopus cell line and Tandem Affinity Purification

A6 cells, derived from *Xenopus* kidney epithelium (ATCC: CCL-102) were maintained in XTC media. Cells were transfected with the plasmids pCS2 + TAP-LL-CA (constructs details can be found in Supplementary Methods) using Lipofectamine 2000 following manufacturer’s instructions (Life Technologies). Transfected cells were cultured for 24 h, washed with PBS, stripped by adding cold PBS + 0.5 mM EDTA, centrifuged, pelleted and frozen at −80 °C. TAP was performed following manufacturer’s instructions (InterPlay Mammalian TAP System, Agilent Technologies). The protocol followed for nanoLC/MS/MS can be found in Supplementary Methods.

### Immunoprecipitation and Western-blot analysis

For immunoprecipitation assay pCS2 + MT-LL-CA and pCS2 + MT were constructed by adding a myc tag (MT) into the *Pst*I site of pCS2+-LL-CA and pCS2+. For FRYL immunoprecipitation (Supplementary Fig S1), HEK-293T cells were transfected with plasmids and nuclear extracts were prepared. Briefly, cells were trypsinized and washed twice with cold PBS. Cell pellets were incubated 15 min on ice in 400 ul of buffer hypotonic (buffer A: 10 mM HEPES, pH 7.9, 1.5 mM MgCl_2_, 10 mM KCl, 0.5 mM DTT and protease inhibitor cocktail) (Halt, Pierce). Cells were lysed with 35 ul of 10% NP-40. After brief vortexing and centrifugation at 4 °C, pellets were resuspended in 200 ul of buffer hypertonic buffer (buffer B: 20 mM HEPES, pH 7.9, 420 mM NaCl, 25% glycerol, 1.5 mM MgCl_2_, 0.5 mM DTT and protease inhibitor cocktail) and gently agitated for 45 min at 4 °C. Following centrifugation, the supernatant containing the nuclear fraction was diluted in 600 ul of dilution buffer (20 mM HEPES, pH 7.9, 1.5 mM MgCl_2_, 0.2 mM EDTA, 1.5% NP-40, 0.5 mM DTT and protease inhibitor cocktail). After a brief centrifugation, the supernatant containing the nuclear fraction was assayed for immunoprecipitation. Lysates were incubated o/n at 4 °C with 9E10 Myc antibody (Covance Research Products Inc) and 1 h with A/G-Sepharose beads for 2 h at 4**°**C. Immunocomplexes were washed five times with 20 mM HEPES, pH 7.9, 1.5 mM MgCl_2_, 150 mM NaCl, 0.5% NP-40, 0.5 mM DTT, 0.2 mM EDTA and protease inhibitor cocktail, resuspended in Laemmli sample buffer and run on 8% SDS-PAGE gels. Membranes were blotted with the primary antibodies 9E10 Myc and anti-FRYL (Abcam RRID:AB_10013622) and secondary antibodies from GE Healthcare RRID:AB_772210 and RRID:AB_772206, respectively. Blots in Supplementary Fig S1 were revealed by ECL Western Blotting Substrate (Fisher Scientific, USA).

For paraformaldehyde crosslinked co-immunoprecipitation of FD + LZ and LL-CA in Fig. 1c, HEK-293T cells were co-transfected with pCS2 + 763-HA-FD + LZ and pCS2 + MT-LL-CA using Lipofectamine 2000 (Invitrogen) according to the manufactures directions. Untransfected HEK-293T cells were used to control for non-specific protein binding, and lysis buffer was used in control experiments for antibody-linked magnetic beads. Crosslinking was performed according to^71^ using 2% paraformaldehyde. Co-IP was performed using HA-tagged (Pierce Anit-HA Magnetic Beads # 88836) or Myc-tagged magnetic beads (Pierce Anti-HA Magnetic Beads # 8842) according to the manufactures directions. Mouse-anti-HA-tag 1:1000 dilution (Thermo Fisher RRID:AB_10978021) or Mouse-anti-Myc-tag 1:1000 dilution (Thermo Fisher RRID:AB_2533008) were used for Western-blot analysis. Infrared dye-conjugated secondary antibodies RRID:AB_2651128, RRID:AB_2721181, RRID:AB_2687825 and RRID:AB_2651127 (Li-COR, Lincoln, Nebraska) were used according to the manufacturer’s specifications. Images were captured using the Odyssey CLX system with a resolution of 169um and auto intensities for each channel.

### Xenopus embryos manipulation and microinjection

*Xenopus laevis* embryos were obtained by artificial fertilization, maintained in 0.2X MMR and staged as previously described^72^. TAP-LL-CA mRNA was transcribed *in vitro* and injected in both ventral blastomeres of 4-cell embryos. For *Fry*-depletion studies, 8-cell embryos were injected into one of the V2 blastomeres (1x V2) with 5–20 ng of *Fry-MO* (Gene Tools, LLC). *Fry-MO* sequence and specificity have been previously published^35^. A standard *Random-control* morpholino was injected as a negative control (Gene Tools, LLC). For rescue experiments, 20 ng of *Fry-MO* were co-injected with HA.FD + LZ mRNA (200 pg or 400 pg) into 8-cell embryos 1x V2 (for details on the construct see Supplementary Methods). For mRNA synthesis the construct was linearized with *Not*I and transcribed with T7 (mMESSAGE mMACHINE Ambion). For synergism experiments, 2.5 ng of *Fry-MO* and/or 50 pg of *Lhx1-AS* were injected into 8-cell embryos (1x V2). *Lhx1-AS* synthesis, sequence and specificity have been previously described^13,73^. Morpholinos and/or *lhx1-AS* were co-injected with rhodamine dextran as a lineage tracer. For miRNA sequencing and axis development experiments, 4-cell embryos were injected into both dorsal blastomeres with 20 ng of *Fry-MO* or 400 pg of *Lhx1-AS*. For synthetic miRNA injections followed by *in situ* hybridization, 8-cell embryos were injected (1x V2) with a total of 50 fmol of miRCURY LNA microRNA Mimics (Exiqon). The following mimics were used and were selected for having the same mature miRNA sequence as the ortholog *Xenopus laevis* miRNA: hsa-miR-199a-5p, rno-miR-214-3p, hsa-miR-23b-3p, hsa-miR-27b-3p, and mimic negative control 4.

### *In situ* hybridization and immunostaining

Whole-mount *in situ* hybridization was carried out as previously described^74^. The *lhx1* construct was linearized with *Xho*I. *Fry* construct (Dharmacon) (nt 7974–8665 NM_001110757.1) was linearized with *Sma*I. *Fryl* construct (Quintara Biosciences) (nt 9694–10093 XM_018229770.1) was linearized with *Sac*I. The *pax8* (gift from Tom Carroll), *myoD* and *β1-NaK-ATPase* (gift from Oliver Wessely) constructs were linearized as previously described^11^. The *wt1* construct was linearized with *Sac*I. The *aldh1a2* construct (Dharmacon, DY558471) was linearized with *EcoR*I. All linearized constructs were transcribed with T7 for antisense probe synthesis. For whole-mount immunostaining with 3G8.2C11 (EXRC) and vibratome sections, we followed the protocols previously described^11^. Following *in situ* hybridization for *fryl* embryos were fixed in Bouin’s, dehydrated, embedded into paraffin blocks and sectioned at 15 μm.

### Real-time qPCR analysis

After injection of 8-cell embryos 2x V2 with antisense oligos (50 pg *Lhx1-AS* and/or 2.5 ng *Fry-MO*), embryos were collected at S15 for RNA analysis. The RNA of five embryos was combined and RNA was isolated using the miRVana microRNA Isolation Kit (Ambion). For quantification of mature miRNA expression, miR-199a-3p (hsa-miR-199a), miR-214 (hsa-miR-214), miR-23b (hsa-miR-23b), miR-27b (hsa-miR-27b), miR-24a-3p (hsa-miR-24) and U6 snRNA were reverse transcribed with respective RT primers using TaqMan® MicroRNA Reverse Transcription Kit (Applied Biosystems) on a standard thermocycler. Assays for all small RNAs were conducted utilizing respective TaqMan MicroRNA Assays amplified and analyzed on a iQ5 Multi-Color Real-Time PCR with iQ5 Optical System Software version 2.1 (Bio-Rad).

### MicroRNA sequencing and analysis

Embryos processed for miRNA sequencing were injected as described above and incubated until stage 10.5. Injected embryos with red fluorescence in the dorsal half were selected for RNA extraction. A total of 30 dorsal half embryos at S10.5 were pooled from 3 independent experiments for each sample (*Fry-MO*, *Lhx1-AS* and Uninjected). RNA was isolated using an RNeasy Micro Kit (Qiagen). MiRNA discovery described above as deep sequencing service was performed by LC Sciences (Houston, TX, USA) using the Illumina high-throughput sequencing technology. A small RNA library was generated from the RNA sample using the Illumina Truseq™ Small RNA Preparation kit to capture small RNA with 3′ OH and 5′P modifications. The purified cDNA library was used for cluster generation on Illumina’s Cluster Station and then sequenced on Illumina GAIIx following vendor’s instruction. Raw sequencing reads (40 nts) were obtained using Illumina’s Sequencing Control Studio software version 2.8 (SCS v2.8) following real-time sequencing image analysis and base-calling by Illumina’s Real-Time Analysis version 1.8.70 (RTA v1.8.70). A proprietary pipeline script, ACGT101-miR v4.2 (LC Sciences), was used for sequencing data analysis. Then, the “impurity” sequences due to sample preparation, sequencing chemistry and processes, and the optical digital resolution of the sequencer detector were removed and the analysis continued with sequenced sequences with lengths between 15 and 32 bases after 3ADT (3′ adapter) cut. The reads were then mapped against pre-miRNA (mir) and mature miRNA (miR) sequences listed in the latest release of miRBase, or *Xenopus laevis* and Chordata genome and grouped. A total of ~24M raw reads with an average of 50.4% mappable reads per sample (standard deviation 3.05) were generated. A total of 1,987 unique miRNAs (known and predicted) were discovered across all samples. Sequencing results with raw and normalized number of reads for each sample have been deposited to GEO (GEO GSE100434).

### Statistical Analysis

All numbers stated in graphs are the composite of multiple experiments, with a minimum of three independent experimental replicates for all animal studies. Statistical analysis for Figs 4–6 and 9 and Supplementary Fig. S5 was performed using Fisher’s exact test, two-tailed. Statistical significance of real time qPCR was tested using a one-tailed paired t-test. For all statistical analyses we used GraphPad Prism version 5.00 (GraphPad Software, www.graphpad.com).

## Electronic supplementary material

Supplementary Information
