# Supplementary material for: The Lhx1-Ldb1 complex interacts with Furry to regulate microRNA expression during pronephric kidney development
Source: Sci Rep. 2018 Oct 30;8:16029. doi: 10.1038/s41598-018-34038-x (PMC6207768; doi:10.1038/s41598-018-34038-x)
Supplement: Supplementary file 1 — Supplementary Information [file 41598_2018_34038_MOESM1_ESM.pdf]

The Lhx1-Ldb1 complex interacts with Furry to regulate microRNA expression during pronephric kidney development

Eugenel B. Espiritu[1,**], Amanda E. Crunk[1], Abha Bais[1], Daniel Hochbaum[2], Ailen S. Cervino[3,4], Yu Leng Phua[5], Michael B. Butterworth[6], Toshiyasu Goto[7], Jacqueline Ho[5], Neil A. Hukriede[1,8,*] and M. Cecilia Cirio[3,4,9,*,**]

Supplementary Figure S1

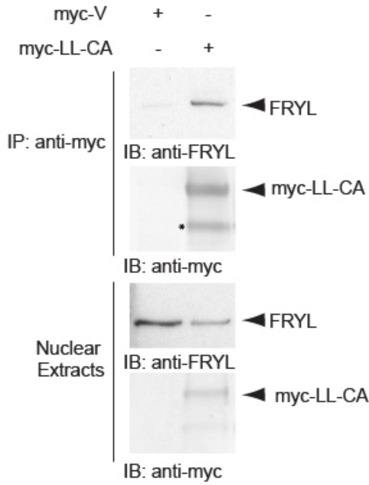

Supplementary Figure S2

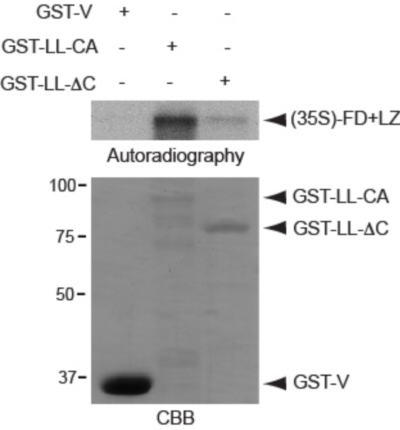

Supplementary Figure S3

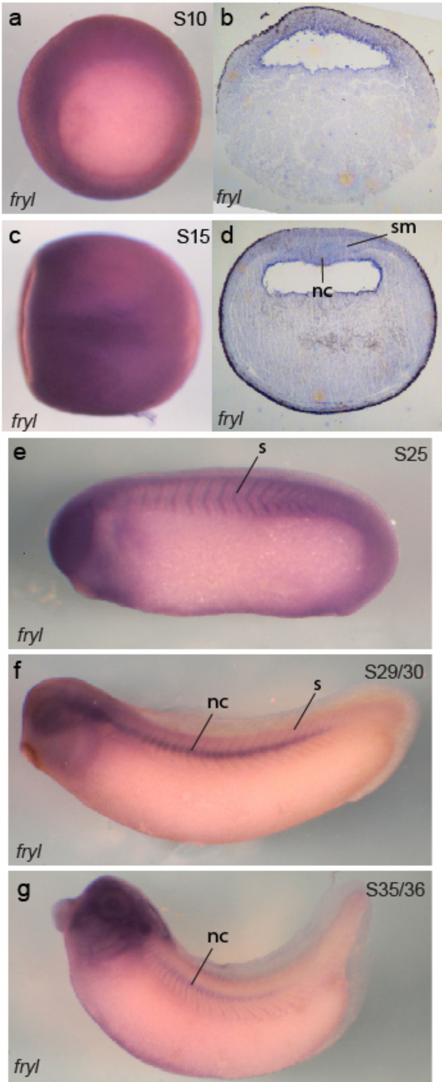

Supplementary Figure S4

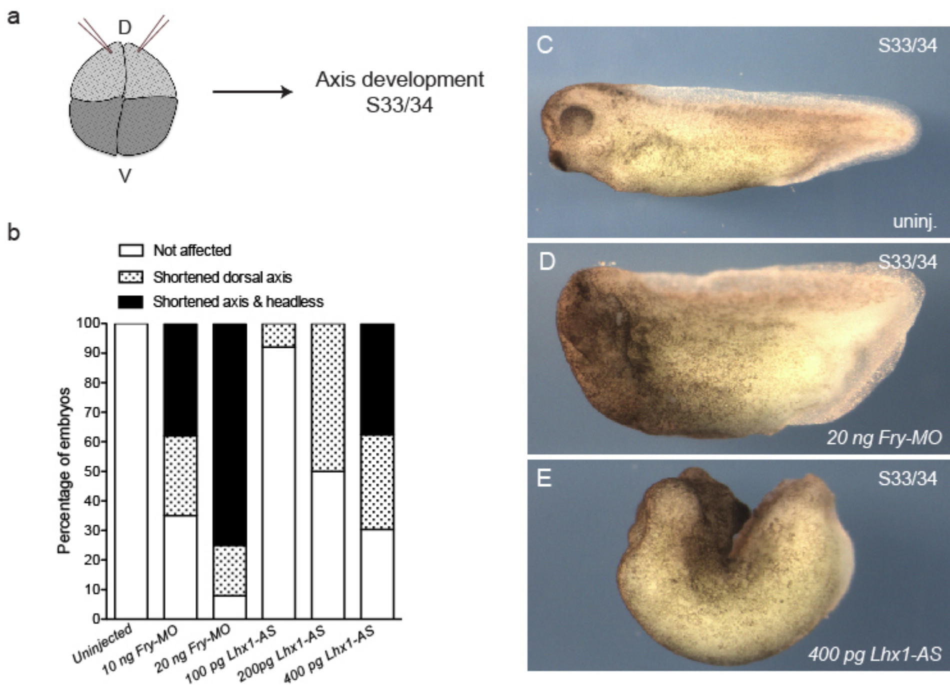

Supplementary Figure S5

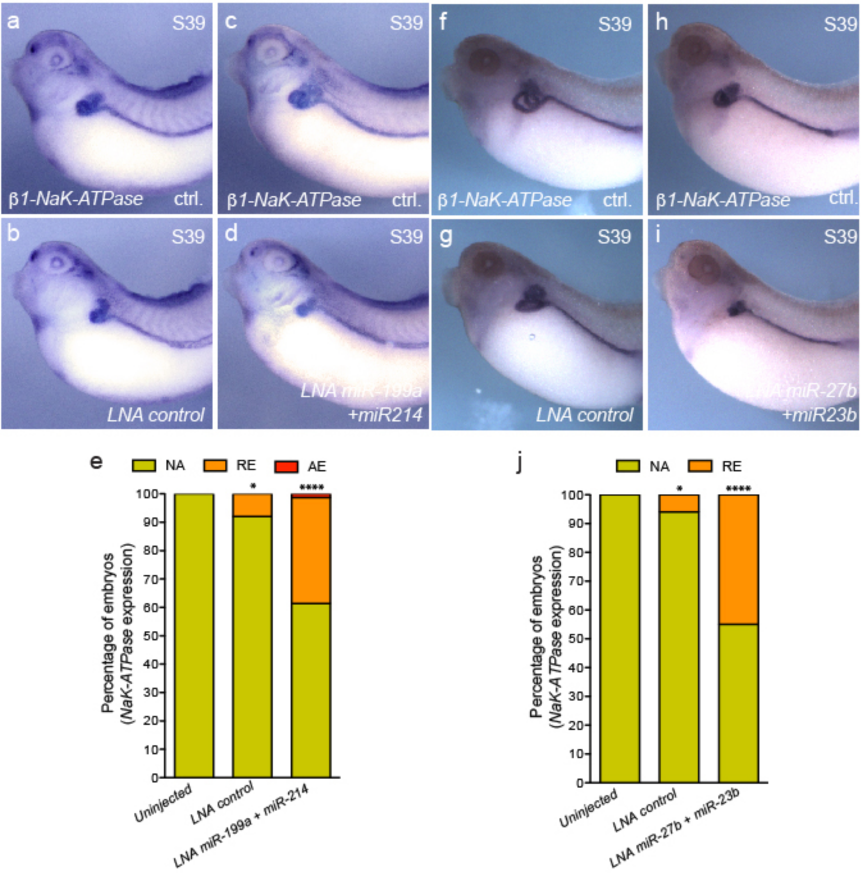

Supplementary Figure S6

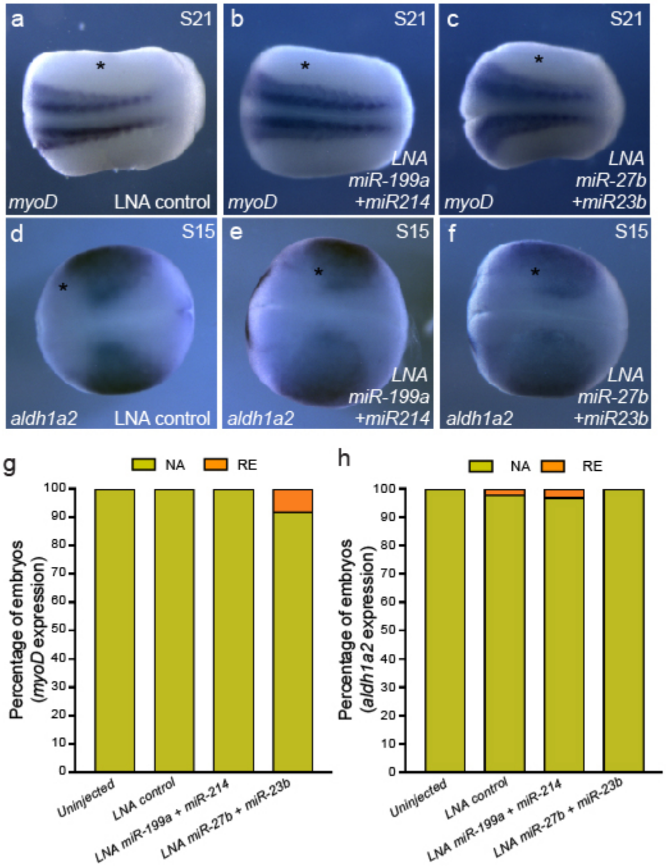

Supplementary Figures Legends

Supplementary Figure S1. Fry-like interacts with the constitutive active Ldb1-Lhx1 complex. Western blot analysis of total nuclear extracts or immunoprecipitated complexes of transfected HEK-293T cells. Cells were transfected either with myc-V or myc-LL-CA, immunoprecipitated (IP) with anti-myc antibody and blotted (IB) either with anti-myc or FRYL antibody. * This band might be a product of degradation of myc-LL-CA.

Supplementary Figure S2. *In vitro* binding assay of the indicated GST-fusion proteins with *in vitro* translated FD+LZ (^{35}S)methionine of Fry. TAP-LL-ΔC lacks the C-terminal conserved regions 2-5 of Lhx1. Coomassie brilliant blue (CBB) staining is shown as input control for the GST-fusion proteins.

Supplementary Figure S3. *Fryl* expression in *Xenopus* embryos. (a, c) Expression of *fryl* in S10 (stage 10) and S15 embryos (stage 15). (b, d) Crossed section of S10 and S15 embryos stained for *fryl*. (e-g) Expression of *fryl* in late neurula S25 and tailbud stages S29/30 and S35/36. Notochord (nc) and somites (s).

Supplementary Figure S4. Induction of axis development defects by depletion of Fry and Lhx1. (a) Representation of the experimental procedure. Both dorsal blastomeres of the 4-cell embryos were injected. (b) Percentage of embryos at S33/34 (stage 33/34) with a shortened axis and/or headless phenotype. Uninjected (N=3, 36), Fry-MO 10 ng (N=4, 110), Fry-MO 20 ng (N=6, 148), Lhx1-AS 100 pg (N=3, 59), Lhx1-AS 200 pg

(N=1, 12), Lhx1-AS 400 pg (N=3, 29). (c-e) Representative embryos of the selected doses of *Fry-MO* and *Lhx1-AS* are shown.

Supplementary Figure. S5. Overexpression of miR-199a/214 and miR-23b/27b reduce the kidney field size. (a-d, f-i) *β1-NaK-ATPase* expression in S21 (stage 21) embryos injected with LNA mimics. (a, b, f and g) Embryos injected with LNA control. (a and f) Uninjected side (ctrl). (b and g) Injected side with LNA control. (c and d) Injected side with LNA miR-199a + miR-214. Uninjected (c) and injected (d) sides of the same embryo. (h and i) Embryos injected with LNA miR-27b + miR-23b. Uninjected (h) and injected (i) sides of the same embryo. (e) Percentage of embryos with abnormal *β1-NaK-ATPase* expression. Uninjected (N=3, 70); LNA ctrl. (N=3, 74); LNA miR-199a + miR-214 (N=3, 92, 39% reduced or absent expression). (j) Percentage of embryos with abnormal *β1-NaK-ATPase* expression. Uninjected (N=3, 96); LNA ctrl. (N=3, 66); LNA miR-27b + miR-23b (N=3, 63, 46% reduced or absent expression). Reduced (RE), absent (AE) or not affected (NA) field of expression. Data in graphs is presented as means. **** p<0.0001, * p<0.05 Fisher's exact test.

Supplementary Figure S6. Overexpression of miR-199a/214 and miR-23b/27b within a V2 blastomere have no significant effects on *myoD* and *aldh1a2* expression. (a-c) *In situ* hybridization of S21 (stage 21) embryos for *myoD*. Asterisk indicates the injected side of the embryo. (d-f) *In situ* hybridization of S15 (stage 15) embryos for *aldh1a2*. (g) Percentage of embryo with reduced (RE) or not affected (NA) *myoD* expression. Uninjected (N=2, 50); LNA ctrl. (N=2, 40); LNA miR-199a + miR-214 (N=2, 52); LNA

miR-27b + miR-23b (N=2, 45, 8% reduced expression). (h) Percentage of embryos with reduced (RE) or not affected (NA) *aldh1a2* expression. Uninjected (N=2, 48); LNA ctrl. (N=2, 44, 2% reduced expression); LNA miR-199a + miR-214 (N=2, 65, 3% reduced expression); LNA miR-27b + miR-23b (N=2, 52).

Supplementary Methods

Plasmid Constructs for Tandem Affinity Purification (TAP)

pNTAP-LL-CA was constructed by PCR amplification and subsequent subcloning of the fragments LL-CA previously described (Dawid, Breen et al. 1998, Hiratani, Mochizuki et al. 2001) into the *Pst*I and *Sal*I sites of the pNTAP vector (InterPlay Mammalian TAP System, Agilent Technologies) in frame with the CBP (Calmodulin-Binding Peptide) and SBP (Streptavidin-Binding Peptide) tags. The fragment CBP-SBP-LL-CA was digested with the enzymes *Sal*I and *Not*I from pNTAP-LL-CA. This fragment was blunted and cloned into pCS2+ previously digested with *Stu*I.

Plasmid Construct for Rescue Experiment

pCS2+763HA.FD+LZ cDNA was made by PCR and cloned into a the modified pCS2+ as *Cla*I and *Avr*II. pCS2+763 plasmid has a *Spe*I and a *Sma*I sites following the *Xba*I sites in the pCS2+ 1st MCS. The following primers were used for the PCR:

Forward: 5'-aaaatcgat**atgtacccatacgatgttccagattacgct**gccagccagcaggactcgggc-3'

Reverse: 5'-aaaacctaggcctgtattcatgggactcgtcttc-3'

Mass Spectrometry (nano LC/MS/MS)

Samples subjected to Tandem Affinity Purification were sent for identification of proteins by nanoLC/MS/MS to MS Bioworks, LLC (Ann Arbor, MI). Briefly, each sample was separated ~1.5 cm on a 10% Bis-Tris Novex mini-gel (Invitrogen), the gel was stained with coomassie and each lane was excised into ten equal sized fragments. The gel pieces were processed and analyzed by nanoLC/MS/MS with a Waters NanoAcquity

HPLC system interfaced to a ThermoFisher Orbitrap Velos Pro. Data were search using a local copy of Macot with the following parameters: Enzyme: Trypsin, Database: Xenbase *X. laevis* and *X.tropicalis*, Peptide Mass Tolerance: 10 ppm, Fragment Mass, Tolerance: 0.8 Da, Max Missed Cleavages: 2. Data were filtered using a minimum protein value of 90%, a minimum peptide value of 50% (Prophet scores) and requiring at least two unique peptides per protein. Results were visualized and validated using Scaffold – Proteome Software 3.3.3. A total of 403 *Xenopus* proteins were detected with two or more peptides at the false discovery rates 0.2% for TAP-LL-CA sample. The mass spectrometry proteomics data have been deposited to the ProteomeXchange Consortium via the PRIDE partner repository with the database identifier PXD006926 and 10.6019/PXD006926.

***In vitro* binding assay**

LL-CA and LL- LL-ΔC (truncated version lacking the C-terminus conserved regions 2-5 of Lhx1) were PCR amplified and cloned into pGEX-4T-3 vector as *Sal*I/*Not*I. GST-fusion proteins were purified from BL21 bacteria. pCS2+763-FD+LZ (Goto et al. 2010) containing the T7 promoter was *in vitro* translated using TNT T7 quick (Promega) and Redivue (^{35}S)methionine (Amersham Biosciences), following manufacturer's directions. Binding assays were performed in binding buffer 50 mM Tris/Cl pH 7.5, 50 mM NaCl, 0.1% Tween-20 and protease inhibitor cocktail (Roche) for 1 h at RT, followed by four washes with binding buffer. Samples were run on 10% SDS-PAGE, fixed with 45% methanol-15% acetic acid solution, soaked with Amplify (Amersham Biosciences), dried and exposed.

MiRNA cluster analysis

The comparison results file from LC Sciences was further processed as follows. We used the R package (R Core Team (2016). R: A language and environment for statistical computing. R Foundation for Statistical Computing, Vienna, Austria. URL https://www.R-project.org/.) mirbase.db (v2.1, miRBase version 19, dated 01 Aug 2012(Griffiths-Jones, Saini et al. 2008)) to identify miRNAs clusters (<10Kb). Further, we selected those miRs whose cluster contains at least 2 detected miRNAs (present in Figure 6 and GEO GSE100434) and at least half of the members are detected (non-zero raw counts) in the uninjected sample. Finally, for each miR in a selected cluster, we evaluated the sign of the fold-change (log base 2) of normalized counts with respect to the uninjected sample to identify clusters that are over-expressed in the absence of a transcription factor and had at least half of the cluster members with non-zero reads in the uninjected sample. This analysis resulted in nine selected clusters (Figure 7).
